# MicroRNA Profile of Lung Tumor Tissues Is Associated with a High Risk Plasma miRNA Signature

**DOI:** 10.3390/microarrays5030018

**Published:** 2016-07-05

**Authors:** Orazio Fortunato, Carla Verri, Ugo Pastorino, Gabriella Sozzi, Mattia Boeri

**Affiliations:** 1 Unit of Tumor Genomics, Department of Experimental Oncology and Molecular Medicine, Fondazione IRCCS Istituto Nazionale dei Tumori, Milan 20133, Italy; orazio.fortunato@istitutotumori.mi.it (O.F.); carla.verri@istitutotumori.mi.it (C.V.); mattia.boeri@istitutotumori.mi.it (M.B.); 2 Unit of Thoracic Surgery, Fondazione IRCCS Istituto Nazionale dei Tumori, Milan 20133, Italy; ugo.pastorino@istitutotumori.mi.it

**Keywords:** microRNA, microarray, liquid biopsy, lung cancer

## Abstract

Lung cancer is the most common cause of cancer deaths worldwide. MicroRNAs (miRNAs) are short, non-coding RNAs that regulate gene expression. Many studies have reported that alterations in miRNA expression are involved in several human tumors. We have previously identified a circulating miRNA signature classifier (MSC) able to discriminate lung cancer with more aggressive features. In the present work, microarray miRNA profiling of tumor tissues collected from 19 lung cancer patients with an available MSC result were perform in order to find a possible association between miRNA expression and the MSC risk level. Eleven tissue mature miRNAs and six miRNA precursors were observed to be associated with the plasma MSC risk level of patients. Not one of these miRNAs was included in the MSC algorithm. A pathway enrichment analysis revealed a role of these miRNA in the main pathways determining lung cancer aggressiveness. Overall, these findings add to the knowledge that tissue and plasma miRNAs behave as excellent diagnostic and prognostic biomarkers, which may find rapid application in clinical settings.

## 1. Introduction

Lung cancer is the most frequent cause of cancer mortality worldwide, accounting for more than 1.4 million deaths per year [1]. Despite improvements in early diagnosis and new therapeutic strategies, the overall five-year survival remains only of 10%–20% [2]. Poor prognosis depends on several features: diagnosis at an advanced stage, tumor heterogeneity, and a limited understanding of lung cancer biology. Advances in genome-wide sequencing and microarray analysis are essential for a better definition of molecular mechanisms enhancing all types of cancer [3], as well as lung cancer [4].

MicroRNAs (miRNAs) are small, non-coding, 22 nt-long, RNAs able to bind complementary sequences of target mRNAs and to induce either their degradation or translational repression [5]. In mammals, miRNAs control the activity of more than 50% of all protein-coding genes [6]. miRNAs are expressed in a tissue specific manner, thereby greatly contributing to cell-type-specific profiles of protein expression. miRNAs potentially target hundreds of different mRNAs, thus regulating a wide variety of cellular processes [7]. To date, more than 1000 human miRNAs have been found in the genome, and a considerable number of them were found differentially-expressed in cancer cells compared to normal cells [8,9]. miRNAs can also be detected in biological fluids, such as plasma or serum, and can serve as circulating biomarkers [10,11]. They can be secreted via extracellular vesicles, such as exosomes [12], or by protein-miRNA complexes, such as high-density lipoprotein (HDL) or Argonaute2, protecting them from plasma RNase digestion [13]. So far, several miRNA-based liquid biopsy tools have been identified for cancer diagnosis and prognosis [14].

In previous studies we identified miRNA signatures with diagnostic and prognostic value in tumor, normal lung tissue, and plasma samples collected from patients enrolled in low-dose computed tomography (LDCT) screening trials [15,16]. In particular, we described a circulating miRNA signature classifier (MSC) able to identify lung cancer with more aggressive features. The MSC is composed by reciprocal ratios among 24 miRNAs, stratifying patients in three different groups: high, intermediate, and low, according to the risk of developing lethal lung cancer [17]. The 24 circulating miRNA were found to be involved in pathways regulating cellular aging, bronchi-alveolar and hematopoietic stem cells renewal, cell survival, and induction of angiogenesis [16]. In addition, among these, miR-660 was demonstrated to possess a therapeutic role in lung cancer cells and in vivo models [18].

In order to investigate if altered circulating miRNA levels associated with prognosis may reflect molecular changes at the level of the tumors, the miRNA expression profile of lung cancer tissues from 19 patients were compared according to the respective MSC risk level. A pathway enrichment analysis was further performed in order to define the biological processes regulated by identified miRNAs.

## 2. Materials and Methods

### 2.1. Patients’ Characteristics

Plasma and tumor tissue samples were collected from 19 patients with a lung tumor detected during the LDCT screening trial performed at the Istituto Nazionale dei Tumori—Istituto Europeo di Oncologia (INT-IEO) [19]. Overall, between 2000 and 2005, a total of 1035 subjects were enrolled and 41 (4%) developed lung cancer during the first five years of screening. The median age was 58 years (range 50–84), 739 (71%) were men, average tobacco consumption was 26 cigarettes daily for 37 years (median pack/years = 40), and 14% were former smokers. Both tissue and plasma specimens were collected from 19 patients at the time of surgery. All samples were obtained according to the Internal Review and the Ethics Boards of the Istituto Nazionale Tumori of Milan and all patients provided informed consent.

### 2.2. MicroRNA Profiling in Plasma Samples

Plasma samples were collected and RNA isolated as previously described [16,20]. Briefly, using mirVana PARIS Kit (Thermo Fisher Scientific, Waltham, MA, USA) total RNA was extracted from 200 μL plasma samples and eluted in 50 µL of buffer. MicroRNA expression was determined using the Megaplex Pools Protocol on microfluidic card type A (Thermo Fisher Scientific). For each microfluidic card (sample), the Ct of every miRNA was determined using the program SDS 2.2.2 (Thermo Fisher Scientific) and setting a threshold of 0.2 and a manual baseline from 3 to 18 cycles. The MSC algorithm was defined as previously described [15,16,20]. Briefly, since no housekeeping miRNAs were found in plasma samples, the MSC was composed by reciprocal ratios among 24 miRNAs differentially expressed between lung cancer patients and healthy individuals. The miRNAs composing the MSC are: miR-101-3p, miR-106a-5p, miR-126-5p, miR-133a, miR-140-3p, miR-140-5p, miR-142-3p, miR-145-5p, miR-148a-3p, miR-15b-5p, miR-16-5p, miR-17-5p, miR-197-3p, miR-19b-3p, miR-21-5p, miR-221-3p, miR-28-3p, miR-30b-5p, miR-30c-5p, miR-320a, miR-451a, miR-486-5p, miR-660-5p, and miR-92a-3p.

### 2.3. MicroRNA Profiling in Tumor Tissue Samples

Total RNA was extracted from fresh-frozen tumor samples available in the biobank of our institution, as previously described [16]. MiRNA labeling and hybridization was performed using 5 μg of total TRIzol (Thermo Fisher Scientific) extracted RNA. The miRNA microarray (Ohio State University Comprehensive Cancer Center, version 2.0, Columbus, OH, USA) used contained probes for 460 mature miRNAs and 167 miRNA precursors spotted in quadruplicate with annotated active sites selected for oligonucleotide design. Hybridization signals were detected with streptavidin–Alexa-647 conjugate, and scanned images (Perkin-Elmer ScanArray XL5K Scanner) were quantified using the GeneSpring software version 7.2 (Silicon Genetics, Redwood City, CA, USA).

### 2.4. Statistical and Bioinformatics Analyses

On the microarray chips, after background subtraction and data transformation (to convert any negative value to 0.01), the average value of the four spots was normalized using a per-chip 50th percentile method that normalizes each chip on its median (data available as Table S2). Class comparison analyses were performed using BRB ArrayTools 4.2.1 software [21]. MicroRNAs differentially expressed between two classes were considered significant at the nominal 0.01 level of the univariate test based on 10,000 random permutations. Survival curves were estimated using the Kaplan-Meier method and were compared by the log-rank test [22].

### 2.5. Pathways Enrichment Analysis

Pathway enrichment analysis was performed considering only mature miRNA differentially expressed using both DIANA-mirPath V.2 and miRWalk2.0 online softwares [23,24]. For mirPath analysis, gene targets were predicted by microT-CDS and the results were merged using the pathway union mode with false discovery rate (FDR) correction, which calculate the merged *p*-value of all miRNAs for each pathway by applying Fisher’s meta-analysis method. Pathways were considered significant with *p*-value < 0.01 and microT threshold = 0.8. For miRWalk analysis, predicted targets identified by miRWalk or Targetscan, and Kyoto Encyclopedia of Genes and Genomes (KEGG) pathways were considered significant having adjusted *p*-values < 0.05 after FDR correction.

## 3. Results

### 3.1. Patients’ Characteristics and Prognostic Value of the miRNA Signature Classifier (MSC)

**Figure 1:**
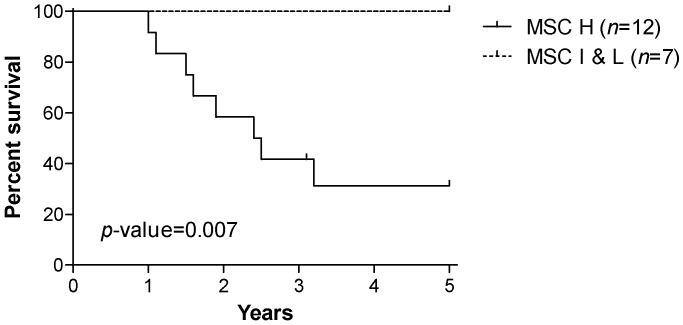
Kaplan Meier curves for 19 lung cancer patients in strata of the miRNA signature classifier (MSC) level of risk: high (H) vs. Iintermediate and low (I and L). *p* for log rank test.

Tumor specimens were collected at the time of surgery from 19 lung cancer patients enrolled in the INT-IEO LDCT-screening trial performed in our institute. Patients were all heavy smokers with a pack-years (PY) index higher than 20 and older than 50 years. The majority of tumors were adenocarcinoma (ADC, 73.7%), while 15.8% squamous cell carcinoma (SCC) and 10.5% other tumors. As expected in a screening-detected tumors population, stage I were more frequent (63.2%) than the higher stages (36.8%), and the 57.9% of patients was still alive after five years of follow-up. Only one patient was censored after 3.1 years. From the same subjects, plasma samples were also available to perform the MSC test: 12 (63.2%) patients resulted high, six (31.6%) intermediate, and one (5.3%) low risk MSC (Table 1). The five-year overall survival (OS) for the 12 patients with high MSC risk level was 31.3%, while for the seven patients with intermediate or low MSC risk levels was 100% (*p*-value = 0.007, Figure 1).

### 3.2. MicroRNA Expression in Tumor Tissue Associated with MSC Risk Level

MicroRNA profiles of the 19 lung tumor tissues were analyzed using the microarray x-platform (Ohio State University Comprehensive Cancer Centre, version 2.0). By stratifying patients in MSC high versus intermediate and low risk level, class comparison analyses showed that the expression of 11 mature miRNAs and six miRNA precursors were significantly different at the nominal 0.01 level of the univariate test (Table 2). In particular, hsa-mir-520e, hsa-mir-518c-5p, and all six miRNA precursors were, resultingly, downmodulated in the high risk group, while hsa-mir-329-3p, hsa-mir-302d-3p, hsa-mir-520f, hsa-mir-511-5p, hsa-mir-509-3p, hsa-mir-519a-3p, hsa-mir-521, hsa-mir-520h, and hsa-mir-499a-5p were overexpressed.

Interestingly, by stratifying patients according to survival status at five years, only two mature miRNAs (hsa-mir-499a-5p and hsa-mir-429) and one miRNA precursor (hsa-mir-212-prec) were found differentially expressed (Supplementary Table S1), being only mir-499a-5p in common with the MSC related signature.

### 3.3. Pathway Enrichment Analysis

A pathway enrichment analysis was performed using the MirPath software and the 11 mature miRNAs. As reported in Table 3, important pathways contributing to tumor aggressiveness were regulated by at least four of these miRNAs. Among these, the following pathways were observed: proliferation pathways, such as *MAPK* signaling pathway (regulated by six miRNAs) and the *ErbB* signaling pathway (by four miRNAs); pathways associated with epithelial to mesenchymal transition (EMT), such as the regulation of the actin cytoskeleton (by six miRNAs) and the *TGF-β* signaling pathway (by four miRNAs) involved in cancer cell dissemination and metastasis formation; the cell survival *PI3K-Akt* signaling pathway (by five miRNAs) and in general pathways associated with non-small cell lung cancer (NSCLC, by four miRNAs), including the *epithelial growth factor receptor* (*EGFR)* signaling.

**Figure 2:**
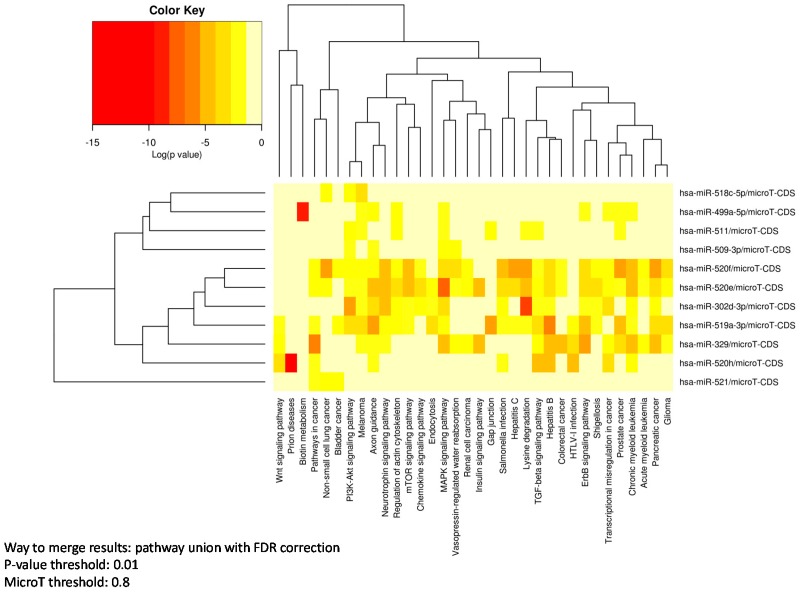
Heat map of Kyoto Encyclopedia of Genes and Genomes (KEGG) pathway enrichment analysis using DIANA-mirPath V.2 and considering 11 mature miRNAs differentially expressed between lung cancer patients with high and intermediate/Low risk miRNA signature classifier (MSC).

In particular, hsa-mir-302d-3p, hsa-mir-329-3p, hsa-mir-519a-3p, and hsa-mir-520e-f were associated with multiple pathways. On the other hand, other miRNAs were associated with a just a few pathways specific to cancer development: hsa-mir-499a-5p, associated with proliferation signaling, and hsa-mir-511-5p and hsa-mir-520h with EMT (Figure 2). Similar results were also obtained using the miRWalk2.0 prediction software, where pathways in cancer, *MAPK* signaling pathway, non-small cell lung cancer, Wnt signaling pathway, and the *ErbB* signaling pathway were significantly (*p* < 0.05) regulated by at least four of these 11 mature miRNAs (Supplementary Figure S1).

## 4. Discussion

Gaining insight into the molecular changes that underlie aggressive lung cancers is of critical clinical relevance. The purpose of this work was to investigate in a subset of CT-detected lung cancer patients the association between plasma miRNA risk profile and their miRNA expression profile in tumor tissue.

We previously demonstrated that a plasma miRNA signature classifier (MSC), stratifying individuals undergoing lung cancer screening into three risk levels (high, intermediate, and low), predicted diagnosis and the overall survival (OS). Moreover, a high-risk miRNA circulating profile was demonstrated to identify malignancy and aggressive disease [15,17].

Analysis of the tissue miRNA profile of lung cancer patients according to prognosis showed differences in only three miRNAs, suggesting high heterogeneity of tumor samples within the two groups. On the other hand, when stratification was performed on the basis of their plasma miRNA risk level, a panel of 11 mature miRNAs and six differentially-expressed miRNA precursors were observed. The different results obtained by miRNA profiles suggest the clinical relevance of molecular stratification in identifying more homogenous subgroup of patients, for a more accurate patient management.

Interestingly, none of the 17 cancerous tissue miRNAs belong to the 24 plasma miRNA panel composing the MSC, supporting the hypothesis that circulating miRNAs are mostly contributed from tumor-surrounding changes, strongly affected by smoking carcinogen exposure, permissive for the developing of an aggressive disease. Accordingly, time dependency analysis showed that the MSC risk level was already positive up to two years before disease detection by LDCT [15]. Moreover, even after cancer removal, relapsing patients retained an elevated MSC level, reflecting the persistence of a host/microenvironment–related risk profile [17].

Published studies showed that mir-499a-5p is a tumor suppressor miRNA by targeting the *VAV3* gene and its reduction correlate with poor clinical outcome in NSCLC. Experimental data show that mir-499a-5p overexpression induces apoptosis, and inhibits cell proliferation in vitro and NSCLC metastasis in vivo [25]. Mir-329, by targeting oncogenic *MET*, reduces cell proliferation, migration, invasion, and promotes apoptosis in lung cancer cell lines [26]. Mir-520e is involved in the *NIK/p-ERK1/2/NF-κB* signaling in hepatocarcinogenesis having as direct target *NIK* gene [27], while mir-519a acts in *STAT3* pathways and is correlated with poor outcome in glioblastoma [28]. Finally, mir-302d-3p is a member of the mir-302/367 cluster that has a role in the regulation of cell signaling pathways involved in the cell cycle and inducing pluripotent stem cells [29,30].

The analysis of pathway enrichment regulated by these miRNAs displayed involvement of relevant biological processes, such as proliferation, growth factor receptor signaling, signal transduction, cell survival, dissemination, and metastasis; features that are altered in the more common cancers, and particularly in lung cancer.

## 5. Conclusions

These results indicate that the biological trait of lung tumors with a high-risk plasma miRNA profile is enriched for features related to disease aggressiveness, supporting the established prognostic role of plasma MSC classification in lung cancer patients enrolled in screening trials. Overall, these findings add to the knowledge that tissue and plasma miRNAs behave as excellent diagnostic and prognostic biomarkers which may find rapid application in clinical settings.

## Figures and Tables

**Table 1:** Clinical-pathological characteristics of 19 low-dose computed tomography (LDCT)-detected lung cancer cases of the Istituto Nazionale dei Tumori—Istituto Europeo di Oncologia (INT-IEO) screening trial with available plasma samples for the miRNA signature classifier (MSC) test.

Clinico-Pathological Charachteristics	Trial INT-IEO *N* = 19	
Gender		
Male	12	(63.2%)
Female	7	(36.8%)
Age (years)	57.5 ± 5.6 (s.d.)	
Smoking habit (Pack-Years index)	60.3 ± 23.8 (s.d.)	
Histotype		
ADC (adenocarcinoma)	14	(73.7%)
SCC (squamous carcinoma)	3	(15.8%)
other	2	(10.5%)
Stage		
Ia-Ib	12	(63.2%)
II-III-IV	7	(36.8%)
Status at 5 years		
Alive	11	(57.9%)
Dead	8	(42.1%)
MSC risk level		
High	12	(63.2%)
Intermediate & Low	7	(36.8%)

**Table 2:** Class comparison analysis of microRNA expression in tumor tissue of lung cancer patients stratified according to MSC risk level: high (H) vs. Intermediate (I) and low (L).

MicroRNA	Parametric *p*-Value	Permutation *p*-Value	Geom Mean of MSC H Patients	Geom Mean of MSC I & L Patients	Fold-Change
hsa-mir-210-prec	0.0003	9.00 × 10^‒4^	782.18	1474.27	0.53
hsa-mir-520e	0.0003	8.00 × 10^‒4^	382.79	528.74	0.72
hsa-mir-520h	0.0006	4.00 × 10^‒4^	712.74	485.72	1.47
hsa-mir-7-2-prec	0.0009	0.0011	762.86	1107.25	0.69
hsa-mir-329-3p	0.0017	0.0016	299.35	277.83	1.08
hsa-mir-520f-3p	0.002	0.0016	304.94	274.83	1.11
hsa-mir-511-5p	0.002	8.00 × 10^‒4^	339.84	306.75	1.11
hsa-mir-521	0.0021	5.00 × 10^‒4^	746.92	525.49	1.42
hsa-mir-15a-prec	0.0032	0.0031	390.85	489.14	0.8
hsa-mir-518c-5p	0.004	0.0055	353.1	418.94	0.84
hsa-mir-147-prec	0.005	0.0049	401.67	457.29	0.88
hsa-mir-302d-3p	0.0054	0.0038	299.31	274.01	1.09
hsa-mir-499a-5p	0.0058	0.0049	1726.93	1028.1	1.68
hsa-mir-125a-prec	0.0062	0.0055	364.76	435.28	0.84
hsa-mir-138-2-prec	0.0067	0.008	893.27	1298.2	0.69
hsa-mir-519a-3p	0.0068	0.0036	2117.4	1501.89	1.41
hsa-mir-509-3p	0.0099	0.0065	1204.47	908.23	1.33

Type of univariate test used: two-sample *t*-test; permutation *p*-values for significant miRNAs were computed based on 10,000 random permutations; nominal significance level of each univariate test.

**Table 3:** List of Kyoto Encyclopedia of Genes and Genomes (KEGG) pathways regulated by at least four mature miRNAs differentially expressed between lung cancer patients with high and intermediate/low risk MSC. DIANA-mirPath V.2.

KEGG Pathway	*p*-Value	#Genes	#miRNAs
*MAPK* signaling pathway	3.66 × 10^‒15^	51	6
Regulation of actin cytoskeleton	8.99 × 10^‒9^	40	6
Hepatitis B	1.68 × 10^‒14^	35	5
Axon guidance	3.39 × 10^‒14^	22	5
Chronic myeloid leukemia	5.98 × 10^‒13^	24	5
*PI3K-Akt* signaling pathway	1.49 × 10^‒12^	67	5
Pancreatic cancer	2.24 × 10^‒11^	23	5
Transcriptional misregulation in cancer	5.40 × 10^‒9^	34	5
*ErbB* signaling pathway	1.35 × 10^‒11^	25	4
Neurotrophin signaling pathway	2.21 × 10^‒11^	23	4
*TGF-beta* signaling pathway	1.34 × 10^‒9^	17	4
Non-small cell lung cancer	3.53 × 10^‒8^	15	4

Way to merge results: pathway union with FDR correction; *p*-value threshold: 0.01; MicroT threshold: 0.8.

## Supplementary Materials

The supplementary materials are available online at http://www.mdpi.com/2076-3905/5/3/18/s1.
