# Supplementary material for: MicroRNA Profile of Lung Tumor Tissues Is Associated with a High Risk Plasma miRNA Signature
Source: Microarrays (Basel). 2016 Jul 5;5(3):18. doi: 10.3390/microarrays5030018 (PMC5040965; doi:10.3390/microarrays5030018)
Supplement: Supplementary file 1 [file microarrays-05-00018-s001.zip › microarrays-127562-supplementary material.pdf]

Supplementary Materials: MicroRNA Profile of Lung Tumor Tissues Is Associated with a High Risk Plasma miRNA Signature

Orazio Fortunato [1], Carla Verri [1], Ugo Pastorino [2], Gabriella Sozzi [1,*] and Mattia Boeri [1]

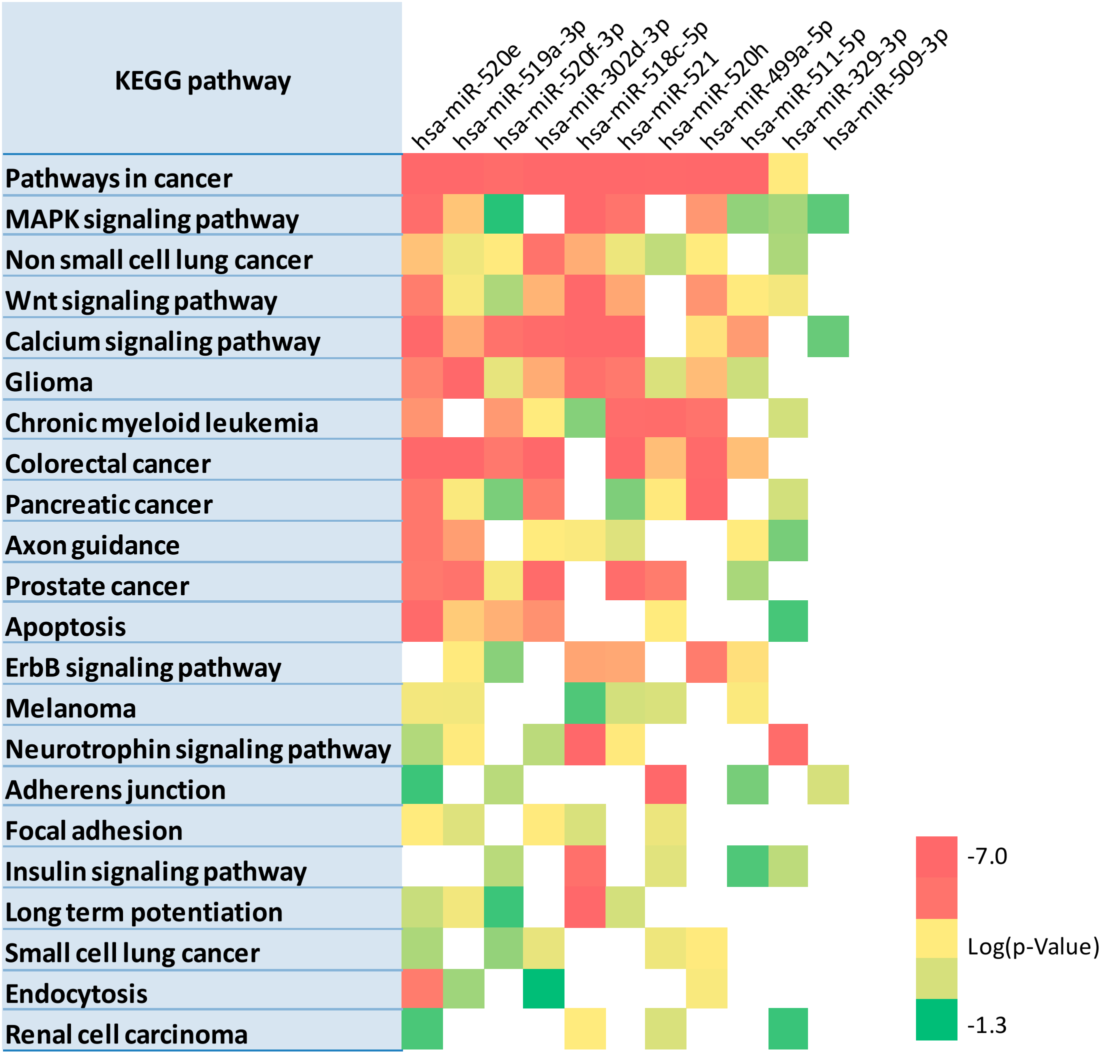

Figure S1. Heat map of KEGG pathway enrichment analysis using miRWalk2.0 and considering 11 mature miRNAs differentially expressed between lung cancer patients with high and intermediate/low risk MSC. Adjusted *p*-value < 0.05 after FDR correction.

Table S1. Class comparison analysis of microRNA expression in tumor tissue of lung cancer patients stratified according to status at five years.

MicroRNA	Parametric *p*-Value	Permutation *p*-Value	Geom Mean of Dead Patients	Geom Mean of Alive Patients	Fold-Change
hsa-mir-499a-5p	0.0058	0.0058	1912.49	1152.67	1.67
hsa-mir-212-prec	0.0066	0.0084	1692.4	1241.48	1.37
hsa-mir-429	0.0079	0.0074	1349.96	910.32	1.49

Type of univariate test used: two-sample *t*-test. Permutation *p*-values for significant miRNAs were computed based on 10,000 random permutations. Nominal significance level of each univariate test: 0.01.
